# IL-6 and IFNγ play a role in fatal cases of 5N1 influenza in children

**DOI:** 10.1186/cc11740

**Published:** 2012-11-14

**Authors:** A Alam, H Jusuf, CB Kartasasmita, D Setiabudi, S Sudarwati, DA Wulandari, AU Suardi, DH Somasetia

**Affiliations:** 1 Medical Faculty Universitas Padjadjaran, Bandung, Indonesia

## Background

Fatal human critical cases associated with influenza A subtype H5N1 have been documented in Bandung, Indonesia. Of four children, three died. We determined the level of cytokines and chemokines in those patients.

## Methods

The Luminex method was used to look for the profile of cytokine and chemokine gene expression induced by H5N1 influenza virus from patient's serum.

## Results

We found that H5N1 influenza virus in the dead children was a more potent inducer of IL-6, the level being higher (17.00, 74.31, 85.75) than in the one child who survived (4.78). The IFNγ level of the fatal causes was also higher (21.43, 31.75, 384.38) than in the one child who recovered (5.51). This suggested that a cytokine storm may play a role in the pathogenesis of fatal H5N1 cases.

## Conclusion

The H5N1 influenza A virus is a potent inducer of proinflammatory cytokines and chemokines. This hyperinduction of cytokines may be relevant to mortality of children with H5N1 infection.

